# Investigating the Cellular Effects of GALC Dosing in Enzyme Replacement Therapy for Krabbe Disease Supports the Role of Nanomedicine

**DOI:** 10.1002/adbi.202500147

**Published:** 2025-07-01

**Authors:** Ambra Del Grosso, Sara Carpi, Laura Colagiorgio, Miriam De Sarlo, Mariacristina Gagliardi, Marco Cecchini

**Affiliations:** ^1^ Istituto Nanoscienze – CNR Pisa, Piazza San Silvestro 12 Pisa 56127 Italy; ^2^ Laboratorio NEST Scuola Normale Superiore Piazza S. Silvestro 12 Pisa 56127 Italy; ^3^ Department of Health Sciences University ‘Magna Græcia’ of Catanzaro Catanzaro 88100 Italy

**Keywords:** autophagy modulation, enzyme replacement therapy, eukaryotic recombinant enzymes purification, galactosylceramidase (GALC), globoid cell leukodystrophy, Krabbe disease, nanomedicine, Twitcher mouse

## Abstract

Krabbe disease (KD) is a lysosomal storage disorder characterized by severe neurodegeneration and demyelination. It is caused by mutations in the galactosylceramidase (GALC) gene, leading to the accumulation of psychosine, a neurotoxic metabolite. This study presents an optimized workflow for the production and characterization of recombinant murine GALC (rm‐GALC) from HEK293T cells, aiming to improve the feasibility of enzyme replacement therapy (ERT) for KD. An affinity chromatography protocol is refined to purify His‐tagged rm‐GALC, followed by buffer exchange and concentration steps to produce a stable and active enzyme suitable for subsequent in vitro applications. The purified rm‐GALC is characterized for enzymatic activity, purity, and stability using SDS‐PAGE, immunoblotting, and dynamic light scattering (DLS). In vitro assays reveal dose‐dependent enzymatic activity recovery in KD primary cells upon rm‐GALC administration, with no adverse effects on cell viability up to the physiological GALC dose. Additionally, GALC treatment at the physiological dose restored autophagic function in KD cells, as shown by LC3 and p62 marker analyses, confirming its compatibility with lysosomal‐autophagic pathways. Conversely, supra‐physiological GALC administration resulted in decreased viability and autophagy impairment. Finally, the feasibility of loading GALC into a polymeric nanovector based on stabilized reverse micelles is investigated. These findings highlight the critical importance of precise GALC dose regulation in developing a safe and effective enzyme replacement therapy (ERT) strategy for Krabbe disease (KD), further supporting the potential of a nanovector‐mediated ERT approach.

## Graphical abstract

A detailed workflow for recombinant GALC production and characterization is presented to support enzyme replacement therapy for Krabbe disease. In vitro assays demonstrate that physiological GALC doses restore enzymatic activity and autophagic flux without affecting cell viability, whereas higher doses impair autophagy and reduce viability. Finally, GALC is successfully encapsulated into stabilized reverse micelles, enabling nanovector‐mediated delivery.

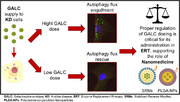

## 1 Introduction

Krabbe disease (KD), also known as globoid cell leukodystrophy, is a rare and severe autosomal recessive lysosomal storage disorder caused by mutations in the galactosylceramidase (GALC) gene. This gene encodes for the protein galactosylceramidase (GALC), a lysosomal enzyme essential for the breakdown of two key sphingolipids: galactosylceramide, a major component of myelin, and psychosine (PSY), a cytotoxic metabolite.^[^ ^1^ ^]^ The degradation of these molecules is critical for maintaining normal myelin turnover, ensuring proper nerve function, and preserving lysosomal homeostasis. When GALC is deficient or dysfunctional, PSY accumulates to toxic levels, leading to widespread demyelination, oligodendrocyte loss, and neuroinflammation.^[^ ^2^ ^]^

Clinically, KD is characterized by progressive motor dysfunction, cognitive decline, and, in its most severe infantile form, rapid neurodegeneration and early mortality. The devastating nature of KD highlights the urgent need for effective therapeutic strategies that can address the underlying enzymatic deficiency and mitigate its downstream effects.^[^ ^3^ ^]^

Currently, therapeutic options for KD are limited and largely palliative. Hematopoietic stem cell transplantation has been used with some success in slowing disease progression, particularly when performed before symptom onset. However, it is not curative and carries significant risks, including complications from immune suppression and graft‐versus‐host disease.^[^ ^3^ ^]^ Gene therapy has shown promise in preclinical studies^[^ ^4^ ^]^ and early clinical trials, offering the potential for sustained GALC expression. Two clinical trials based on gene therapy are currently ongoing to evaluate the efficacy of GALC gene delivery via adeno‐associated vectors in KD patients (FBX‐101 and PBKR03; ClinicalTrials.gov Identifier: NCT04693598 and NCT04771416).

However, it should be noted that while the NCT04693598 trial is currently active but not recruiting, the NCT04771416 trial has been suspended due to changes in the company's strategies. This highlights the challenges and uncertainties in the clinical translation of gene therapy approaches for KD.^[^ ^5^ ^]^

Among these strategies, enzyme replacement therapy (ERT) has emerged as a promising avenue, particularly for other lysosomal storage disorders such as Gaucher, Fabry, and Pompe diseases.^[^ ^6^ ^]^ By supplying functional enzymes to affected tissues, ERT directly addresses the enzymatic deficit. However, translating ERT to KD has been hindered by challenges in producing sufficient quantities of active GALC, ensuring its lysosomal targeting, and achieving effective delivery to the central nervous system (CNS).

Recent advances in nanotechnology have enabled the development of nanocarriers loaded with functional GALC or other lysosomal enzymes, capable of crossing the blood–brain barrier (BBB). These innovative delivery systems have shown promising efficacy in preclinical models of KD^[^ ^7^, ^8^, ^9^ ^]^ and other LSDs,^[^ ^10^, ^11^, ^12^ ^]^ offering a potential solution to overcome the challenges of targeting the CNS.

Advances in recombinant protein production platforms, instead, particularly mammalian expression systems like HEK293T cells, have opened new avenues for the production of therapeutic enzymes.^[^ ^13^ ^]^ Mammalian cells offer the advantage of performing post‐translational modifications critical for lysosomal enzyme functionality, including glycosylation^[^ ^14^ ^]^ and mannose‐6‐phosphate (M6P) tagging for lysosomal targeting.^[^ ^15^, ^16^ ^]^ Despite these technological advancements, the production of recombinant GALC remains challenging due to its complex structure, low natural abundance, and susceptibility to denaturation. These obstacles necessitate the development of robust purification protocols and rigorous characterization of the enzyme's activity, stability, and therapeutic efficacy.

In our earlier work, we efficiently loaded purified rm‐GALC into Angiopep‐2‐targeted polymeric nanoparticles capable of delivering functional rm‐GALC to the brain in Twitcher (TWI) mice, a preclinical model of KD.^[^ ^7^ ^]^ Although those studies demonstrated the therapeutic potential of this approach, a comprehensive biochemical and biophysical characterization of the enzyme was not included. The present study addresses this by providing a detailed and in‐depth pipeline for the production, purification, and validation of rm‐GALC, establishing a solid foundation for future applications in nanoparticle‐mediated delivery and therapeutic testing. This thorough characterization represents an essential step toward the translational development of GALC‐based therapies for KD.

Specifically, we developed an optimized protocol for the production and purification of recombinant murine GALC (rm‐GALC) from HEK293T cells. Using a His‐tagged affinity chromatography approach, we successfully isolated pure and enzymatically active rm‐GALC. The purified enzyme was characterized using a suite of biochemical and biophysical assays to confirm its stability, purity, and functionality.

Subsequently, to evaluate the therapeutic potential of recombinant murine GALC (rm‐GALC), we assessed its ability to restore enzymatic activity and lysosomal function in TWI mouse‐derived fibroblasts, a primary cellular model of KD characterized by complete GALC deficiency.

The TWI mouse, which replicates the key pathological features of KD, including GALC deficiency, PSY accumulation, and progressive demyelination, is the most commonly used model for studying KD and evaluating potential therapeutic strategies.^[^ ^6^, ^17^, ^18^ ^]^

To evaluate the safety profile of rm‐GALC, we assessed its effects on cell viability at both physiological and supra‐physiological doses, uncovering distinct impacts on cell survival rates. Furthermore, we explored the influence of rm‐GALC on the autophagy pathway, which has been previously shown to be dysregulated in KD by our group and others.^[^ ^19^ ^]^ Specifically, prior studies highlighted the accumulation of p62‐tagged molecules in KD cells and neural tissues, highlighting an impairment of the autophagy flux and the inability to efficiently clear “waste molecules.”^[^ ^20^, ^21^, ^22^ ^]^ In this study, we focused on two critical autophagic markers, p62 and LC3, demonstrating a noteworthy dose‐dependent effect of GALC administration on autophagy flux. Finally, we also investigated the suitability of GALC to be loaded into our recently optimized nanovector, based on cross‐linked reversed micelles (stabilized reversed micelles, SRMs).^[^ ^23^ ^]^

Altogether, the findings presented in this paper provide valuable insights into the therapeutic potential of recombinant murine GALC in restoring lysosomal function and modulating autophagy in KD.

## 2 Experimental Section

### 2.1 Animal

Adult wild‐type (WT) and TWI mice were used to establish primary fibroblast cell culture, as described in the following paragraph. TWI heterozygous mice (TWI+/− C57BL6 mice; Jackson Labs), generously provided by Dr. A. Gritti (San Raffaele Telethon Institute for Gene Therapy, Milan, Italy), were used for breeding to generate homozygous TWI mice (TWI−/−, hereafter referred to as TWI for simplicity). The animals were kept under standard housing conditions, and all procedures were conducted according to protocols and ethical guidelines approved by the Ministry of Health (Permit Number: Prot. B4BB8.46; Authorization No. 860/2023‐PR released on October 5, 2023). For genotyping, genomic DNA was extracted from tail clips using Proteinase K digestion followed by genomic DNA purification (EUROGOLD Tissue‐DNA Mini Kit, Euroclone), as previously performed by our group.^[^ ^20^, ^24^ ^]^ The genetic status of each mouse was determined by analyzing the TWI mutation using a fast real‐time PCR‐based protocol recently developed in our laboratory.^[^ ^23^ ^]^ The mouse colony is periodically refreshed with WT Mus musculus C57BL/6 mice to avoid the expression of undesirable genetic traits caused by excessive inbreeding.

### 2.2 Cell Culture and Treatment

#### 2.2.1 Rm‐GALC Overexpressing Cell Culture

Human embryonic kidney 293T cells stably expressing and secreting His6‐tagged murine WT GALC (provided by J. Deane, Cambridge University)^[^ ^14^ ^]^ were initially maintained in 400 mM zeocin selection medium in 150‐mm cell culture dishes. After at least 2 weeks of selection, each dish was transferred to a 1450‐cm^2^ inner surface roller bottle and maintained in complete DMEM [high‐glucose DMEM supplemented with 10% heat‐inactivated fetal calf serum (FCS), 4 mM L‐glutamine, 1% MEM non‐essential amino acids, and 1% penicillin/streptomycin, all from GIBCO‐Life Technologies] at 37 °C, 5% CO₂, and 0.7 rpm. Subsequently, the cells were tested for GALC enzymatic activity in the extracellular medium using the HMU‐*βGal* (see Paragraph 3.3 of Materials and Methods) to confirm sufficient GALC activity before initiating enzyme purification (see Paragraph 3 of the Results). The murine GALC sequence was selected for recombinant enzyme production instead of the human sequence, as the subsequent aim was to restore GALC enzymatic activity in primary GALC‐deficient murine cells derived from Twitcher (TWI) mice.

#### 2.2.2 Primary Fibroblast Cell Cultures

Fibroblast cultures from adult WT and TWI mice were obtained following a protocol established in Dr. Evan Eichler's lab (University of Washington, https://genome.ucsc.edu/ENCODE/protocols/cell/mouse/Fibroblast_Stam_protocol.pdf) as in ref. [25]. The mice were under 6 months old. In brief, after anesthesia, the ears of the mice were removed, rinsed with sterile water, and cut into small fragments. These fragments were collected into an Eppendorf tube and treated with collagenase XI (C7657–100 mg; Sigma–Aldrich), diluted in high glucose Dulbecco's Modified Eagle Medium (DMEM) (approximately 2.5 mg collagenase −320 CDU‐ per mouse). Following a 2‐h incubation at 37 °C, the tube was centrifuged at 200 g for 5 min, the supernatant was discarded, and the pellet was washed with 2 ml of PBS. After another round of centrifugation and supernatant removal, Trypsin‐EDTA 0.05% (59418C‐100ML; Thermo Fisher Scientific) was added to the pellet and incubated for 45 min at 37 °C. The pellet was then centrifuged again, resuspended in complete DMEM [high glucose DMEM supplemented with 10% heat‐inactivated fetal calf serum (FCS), 4 mM L‐glutamine, 1% MEM nonessential amino acids, and 1% penicillin/streptomycin, all from GIBCO‐Life Technologies]. The cells were further dissociated by pipetting up and down with a syringe, plated in 60 mm culture dishes, and incubated at 37 °C in a humidified atmosphere with 5% CO_2_. The next day, the cells were washed and fresh medium was added. Once they reached confluence (after approximately 3–4 days), the cells (passage 0) were washed with 1 ml of PBS and split at a 1:2 ratio. Cells were used for experiments within 10 passages.

#### 2.2.3 Enzyme Administration

For enzyme replacement experiments, WT and TWI fibroblast cells were plated in standard 96‐well cell plates (12500 cell per well). 24 h later medium was replaced with fresh medium containing rm‐GALC at different concentrations (0.0078 U; 0.0775 U; 0.775 U; 3.875 U; 7.75 U; 15.5 U; 38.75 U; 77.5 U; 155 U; enzymatic unit (U) = nmol h^−1^). After 4 h of rm‐GALC incubation medium was removed and the cells were washed three times with 100 µL of PBS. Then, cells were lysed for the enzymatic HMU‐*βGal* assay.

#### 2.2.4 Cell Viability

For viability measurements, we employed the 2‐(2‐methoxy‐4‐nitrophenyl)‐3‐(4‐nitrophenyl)‐5‐(2,4‐disulfophenyl)‐2H‐tetrazolium monosodium salt (WST‐8) assay, following the manufacturer's instructions (Sigma, #96992). Specifically, 100 µL of cell suspension (5000 cells/well) was dispensed into a 96‐well plate and pre‐incubated for 24 h in a humidified incubator at 37 °C with 5% CO_2_. Then, different doses of rm‐GALC (3.875 U; 7.75 U; 15.5 U; 38.75 U; 77.5 U; 155 U; enzymatic unit (U) = nmol h^−1^) were added to the culture media, and the plate was incubated for 24 or 48 h. Prior to use, the CCK‐8 reagent was thawed at room temperature or in a 37 °C water bath. After thawing, 10 µL of CCK‐8 solution was added to each well, taking care to avoid introducing bubbles. The plate was then incubated for 2 h, and the absorbance of each well was measured at 450 nm using a GloMax DISCOVER plate reader (Promega).

### 2.3 Enzymatic Assays

#### 2.3.1 4‐MU‐βGal Assay

The enzymatic activity of purified recombinant murine GALC (rm‐GALC) was measured using the fluorogenic substrate 4‐methylumbelliferyl‐β‐D‐galactopyranoside (4‐MU‐βGal; Figure S1a, Supporting Information). To ensure the assay remained within the linear range of the enzyme activity curve, 1 µL of diluted rm‐GALC (1:100 dilution in storage buffer) was added to 24 µL of assay buffer (50 mM sodium citrate, 125 mM sodium chloride, 0.5% Triton X‐100; pH 4.5), and 25 µL of 1 mM 4‐MU‐βGal substrate. The mixture was incubated for 2 h at 37 °C.

**Figure 1:**
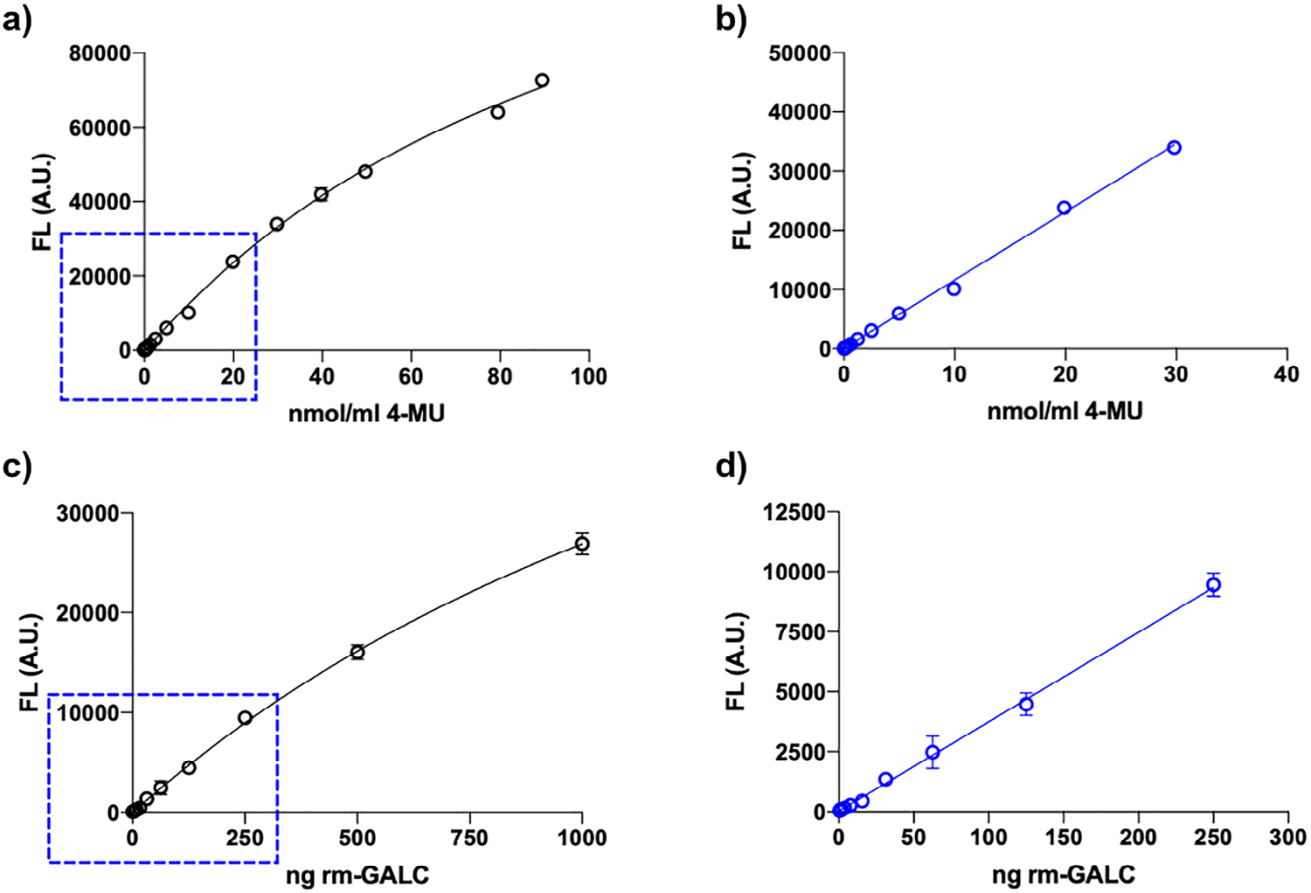
GALC enzymatic assays optimization. a) Graph showing the detected fluorescence (FL) plotted against the nmol of the fluorescent product 4‐MU. b) The linear range for FL versus nmol of 4‐MU selected for accurate extrapolation of enzyme activity from FL measurements is between 0 and 30 nmol mL^−1^ of 4‐MU. c) Graph showing the detected FL plotted against the amount of rm‐GALC in ng. d) The linear range for FL in relation to the amount of rm‐GALC (ng) used in the enzymatic assays spans from 0 to 250 ng of rm‐GALC.

After incubation, the reaction was stopped by adding 150 µL of stop solution (0.5 M glycine, 0.3 M sodium hydroxide; pH 10.0), and the fluorescence of the product was measured using a GloMax Discover Microplate Reader (Promega). Enzymatic activity was calculated as nanomoles of product generated per milligram of protein per hour, using a standard curve derived from fluorescence readings of varying concentrations of 4‐methylumbelliferone (4‐MU; **Figure** **1**b).

The protein concentration of the purified rm‐GALC was determined using the Pierce BCA Protein Assay (Micro BCA Protein Assay Kit, Thermo Fisher Scientific).

#### 2.3.2 HMU‐βGal Assay

To monitor GALC activity in the extracellular medium of HEK293T cells overexpressing GALChis6, we used 6‐hexadecanoylamino‐4‐methylumbelliferyl‐β‐D‐galactopyranoside (HMU‐βGal; Figure S1b, Supporting Information), a fluorescent substrate commonly employed in the clinical diagnosis of KD.^[^ ^26^, ^27^ ^]^ Ten microliters of extracellular medium were added to 20 µL of a 50 mM HMU‐βGal substrate solution and incubated for 2 h at 37 °C.

After incubation, the reaction was stopped with 170 µL of stop solution (0.2 m glycine/NaOH buffer, 0.2% sodium dodecyl sulfate (SDS); pH 10.7; maintained at room temperature to avoid precipitation). The fluorescent product, 6‐hexadecanoylamino‐4‐methylumbelliferone (HMU), was then measured using a GloMax fluorescence reader. GALC activity was expressed as fluorescence intensity (arbitrary units) over the 2‐h incubation for enzymatic assays performed on the extracellular medium.

To assess enzymatic activity in WT and TWI fibroblasts, cells were lysed on ice using radioimmunoprecipitation assay (RIPA) buffer (Sigma–Aldrich) supplemented with protease inhibitors. Cell lysates were sonicated for 4 s at 12 mm intensity. For tissue samples, homogenization was performed in 1.5 ml Eppendorf tubes using appropriate micropestles. Following lysis or homogenization, samples were centrifuged at 15000 g for 25 min at 4 °C, and the supernatants were collected. Protein concentration in the supernatants was determined using the Micro Bicinchoninic Acid (BCA) Protein Assay Kit (Thermo Scientific Pierce). The enzymatic assay for cell and tissue extracts followed the same procedure as described for the extracellular medium, but with a longer incubation time of 17 h. GALC activity was calculated as nanomoles per milligram of protein extract over 17 h, using a standard curve generated from the fluorescence measurements of various concentrations of 4‐methylumbelliferone (4‐MU; Figure 1b). 4‐MU was used for the standard curve instead of HMU due to its high similarity to HMU and significantly lower cost.

### 2.4 Affinity Chromatography Purification

The culture medium is taken from the bottles, and a centrifuge is carried out (1200–1300 rpm, 5 min). The supernatant is filtered through a 0.45 µm filter and incubated overnight at 4 °C at 0.7 rpm together with 5 mL of purification resin (Ni Sepharose excel histidine‐tagged protein purification resin; 17‐3712‐03, GE Healthcare Life Sciences) previously mixed with the binding buffer (sodium phosphate monobasic 20 mm, NaCl 0.5 m, Imidazole 25 mm; pH 7.4) to trigger protein binding. After 12 h, we proceed with the purification by transferring the content into the column and followed by six washings with a column volume of wash buffer (sodium phosphate monobasic 20 mM, NaCl 0.5 M, Imidazole 25 mm; pH 7.4). The washing is a crucial step to obtain a pure and contaminant‐free enzyme. We proceed with elution with a column volume of elution buffer (sodium monobasic phosphate 20 mM, NaCl 0.5 M, Imidazole 500 mM; pH 7.4).

### 2.5 GALC Buffer Exchange and Concentration

The solution containing rm‐GALC is eluted directly into 50 mL falcons containing a 10 kDa cut‐off filter maintained on ice. This is followed by three centrifugations (4000 rpm, 4 °C, 50 min) spaced out by the addition of storage buffer (MES 25 mM, sodium chloride 150 mM, glycerol 20% v/v; pH 5.5) to completely remove the elution buffer and replace it with storage buffer. Finally, 3 (4000 rpm, 4 °C, 50 min) centrifugations are carried out to concentrate the enzymatic solution. The purified enzyme in storage buffer is kept at −20 °C until use.

### 2.6 SDS‐PAGE

Samples from the rm‐GALC purification process were prepared by diluting in Laemmli buffer containing β‐mercaptoethanol (5% final concentration). The samples were denatured by boiling at 95 °C for 5 min, followed by centrifugation at room temperature. The resulting supernatants were used for gel electrophoresis (SDS‐PAGE).

SDS‐PAGE was performed using Bis‐Tris Criterion XT Precast Polyacrylamide Gels (Bio‐Rad, Hercules, CA) in a gel electrophoresis system. Each gel was loaded with 40 µL of the purified samples, alongside a molecular weight marker for reference. Electrophoresis was conducted in 1X running buffer (25 mM Tris, 192 mM glycine, 0.1% SDS) at a voltage of 120–150 V for approximately 1–2 h, until the dye front reached the bottom of the gel.

Following electrophoresis, the gels were stained with Coomassie Brilliant Blue for 2 h to visualize the protein bands. Excess stain was removed by overnight incubation in a de‐staining solution, allowing for clear identification of the protein bands.

### 2.7 Immunoblotting

For Western blot experiments, samples from rm‐GALC purification were prepared by boiling in Laemmli buffer and centrifuging, as described above for SDS‐PAGE experiments. A total of 40 µL of each sample was separated by SDS‐PAGE on 4–12% Bis‐Tris Criterion XT Precast Polyacrylamide Gels (Bio‐Rad, Hercules, CA), following the same procedure as described previously. After electrophoresis, the proteins were transferred onto nitrocellulose membranes using the Trans‐Blot Turbo Transfer System (Bio‐Rad). Immunodetection was performed overnight at 4 °C using an anti‐GALC antibody (ab137750, Abcam). The following day, the membranes were incubated with peroxidase‐linked secondary antibodies (goat anti‐rabbit or mouse IgG‐HRP conjugate, Bio‐Rad) and developed using clarity enhanced chemiluminescent substrates (Bio‐Rad). The chemiluminescent signals were captured using the ChemiDoc XRS+ System with Image Lab Software (Bio‐Rad).

### 2.8 Synthesis of GALC‐Loaded Stabilized Reverse Micelles

The synthesis of empty stabilized reverse micelles (SRMs) was performed as previously detailed in ref. [23]. Subsequently, to load rm‐GALC into SRMs, encapsulation was achieved by maintaining the SRMs dispersed in dimethyl carbonate (DMC) in contact with a rm‐GALC solution (concentration: 5 µg mL^−1^) in the storage buffer previously described (Section 2.5). Specifically, 10 mg of SRMs were dispersed in 150 µL of DMC, and 50 µL of rm‐GALC solution (corresponding to 0.25 µg of enzyme) was then added. The mixture was kept at 4 °C for 48 h under mild stirring. After this period, the non‐miscible top layer (residual rm‐GALC and buffer) was manually removed, and the loaded SRMs in the organic phase were dried under vacuum for 24 h. Finally, dried SRMs were resuspended in 50 µL of a trehalose solution (100 mg mL^−1^) at a concentration of 200 mg mL^−1^. Loading tests were performed in triplicate. To determine GALC activity in SRM samples, 1 µL of SRM was added 24 µL of assay buffer (50 mM sodium citrate, 125 mM NaCl, 0.5% Triton X‐100, pH 4.5), and then 25 µL of 1 mm 4‐MU‐βGal in assay buffer was added. The same assay was performed on the supernatant solution. The enzymatic assay was performed in triplicate for each loaded sample as described in Section 2.3.1.

### 2.9 DLS

Molecular size and aggregation state of GALC and SRMs sizes were measured by dynamic light scattering (DLS) analysis (Zetasizer Nano, Malvern). Samples containing the rm‐GALC solution in DMEM (1‐1.5 µg µL^−1^) or the GALC‐SRMs solution in water (0.1 mg mL^−1^) were added to a quartz cuvette and measured. Data were acquired with a scattering angle of 90. At the end of the measure, raw data were interpolated with a skewed bell‐shaped function to obtain the parameters of interest: peak mode, full width at half maximum (FWHM), and polydispersion index (PDI) by using Excel (Microsoft). Three measures were performed. The z‐potential of GALC‐SRMs was measured on the same sample used for size analysis. The sample was added to a folded‐capillary cell and measured in triplicate.

### 2.10 Immunocytochemistry

WT and TWI fibroblasts were cultured and fixed for 20 min in 4% formaldehyde and 4% sucrose in PBS at room temperature (RT). After fixation, the cells were washed three times with PBS, with each wash lasting 10 min. Following the washes, the cells were incubated overnight at 4 °C in a humidified chamber with primary antibodies: anti‐LC3 (ab48394, Abcam; 1:200) and anti‐p62 (ab56416, Abcam; 1:100), diluted in GDB buffer (0.2% BSA, 0.5% Triton X‐100, 0.8 M NaCl, and 30 mM phosphate buffer, pH 7.4).

The next day, the cells were washed three times with PBS and incubated for 1.5 h at RT with a mixture of secondary antibodies: anti‐rabbit Alexa 647 (A‐31572, Thermo Fisher Scientific) and anti‐mouse Alexa 488 (A‐11029, Thermo Fisher Scientific), both diluted 1:1000 in GDB buffer. After incubation, the cells were washed three times with PBS and once with water to remove any residual salts. Finally, Vectashield mounting medium with DAPI (F6057‐20ML; Sigma–Aldrich) was applied, and the plates were stored at 4 °C until further use.

### 2.11 Confocal Microscopy and Images Analysis

Immunostained cells and brain sections were imaged using a Leica TCS laser scanning confocal microscope, equipped with a 63× oil objective and UV, Ar, HeNe, and He lasers for excitation at wavelengths of 405, 488, and 633 nm, respectively. Confocal images were captured as z‐series stacks with a maximum depth of 10 µm, using step sizes of 0.5–1 µm.

To analyze autophagosomes and p62 aggregates, images were thresholded using the “Threshold” tool in ImageJ (set to 70 for p62 aggregates and 100 for autophagosomes). Binary images were then inverted, and the “Analyze Particles” plugin was applied to count particles. The following parameters were used: “size (micron^2^)” ranging from 1 to infinity, and “circularity” from 0.00 to 1.00. Quantification was performed as described in ref. [20].

All images for analysis were acquired at the same magnification, with consistent confocal settings (pinhole aperture at 1.0 Airy unit, 1024 × 1024 pixels). Z‐stacks were processed in ImageJ (NIH; RRID:SCR_0 03070) using the “z‐project” and “Max intensity” options to generate single images. These processed z‐stacks were then merged using the “Merge Channels” function for multichannel visualization.

### 2.12 Statistical Analysis

Data were obtained from at least three independent experiments (in figure legends, “*n*” refers to the number of experiments performed). For parametric data, either an unpaired two‐tailed Student's t‐test or one‐way ANOVA (followed by Tukey's or Dunnett's multiple‐comparisons test) was applied. The mean values from each experiment were assumed to follow a normal distribution around the true mean. Statistical significance was set at *p* < 0.05.

## 3 Results

### 3.1 Set‐Up of the Workflow for Recombinant Murine GALC Purification

**Figure 2:**
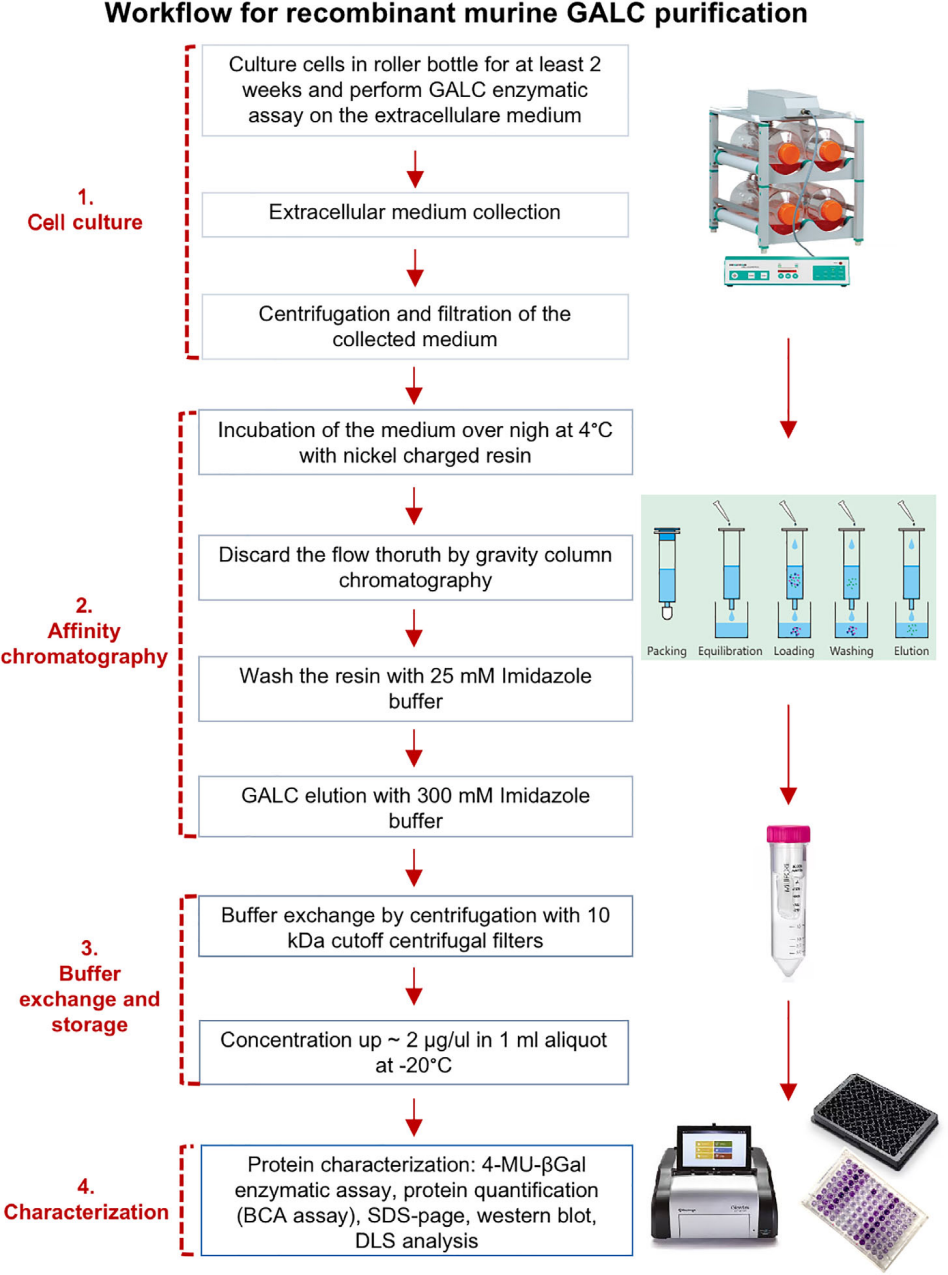
Workflow for recombinant murine Galactosylcermaidase (rm‐GALC) purification. A schematic representation of the purification process for rm‐GALC. Briefly, extracellular media are collected every 14 days from roller bottle cell cultures overexpressing His6‐tagged rm‐GALC. The media is centrifuged, and the supernatant is filtered through a 0.45 µm filter. After overnight incubation with nickel‐charged resin, gravity column chromatography is performed. A 25 mm buffer is used for six washing steps, followed by elution with a 300 mM buffer. The eluted enzyme is then buffer‐exchanged into the appropriate storage buffer, concentrated to approximately 2 µg µL^−1^ with 10 kDa cutoff centrifugal filters, and stored at −20 °C. The enzyme is characterized through enzymatic activity and protein quantification assays, SDS‐PAGE, Western blot, and DLS analysis.

**Figure 3:**
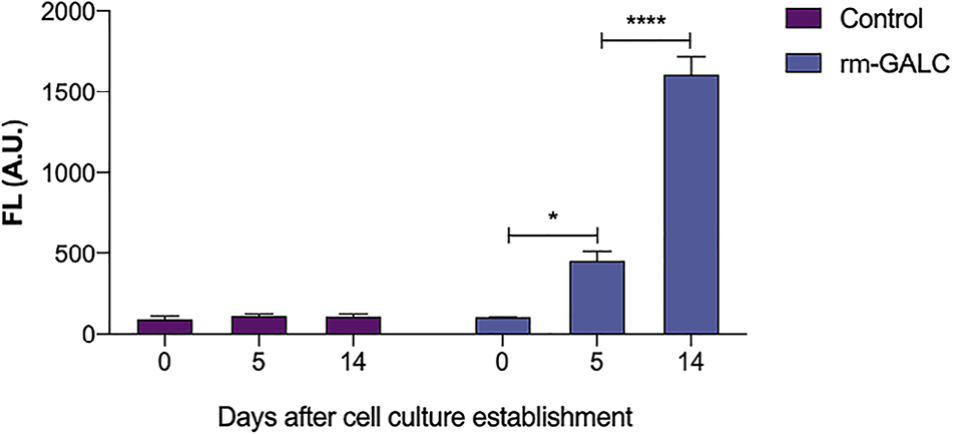
Monitoring of extracellular rm‐GALC activity in roller bottle cell culture. The graph shows results from the HMU‐β‐gal enzymatic assays to measure the concentration of rm‐GALC in the extracellular media of HEK293T cells engineered to overexpress His6‐tagged rm‐GALC. Measurements were taken at three time points: 0, 5, and 14 days after cell culture establishment. Control HEK293T cells were also included in the assays (see the legend). No FL was detected at the days of culture establishment, while a significant increase in FL was observed 5 days later (*p < 0.05; one‐way ANOVA, Dunnett's test), with a peak 14 days later (****p < 0.0001; T2 vs. T0). Values are presented as the mean ± standard error of the mean (SEM). *n* = 3 independent experiments.

We established and optimized a reproducible workflow for the purification of recombinant murine GALC (rm‐GALC) from HEK293T cells (**Figure** **2**). Starting with large‐scale cell culture in roller bottles, this approach allowed for the efficient production of rm‐GALC secreted into the extracellular medium. After 2 weeks of culture, enzymatic activity in the medium was verified via the HMUβ‐gal assay, confirming robust expression of the recombinant enzyme (See Section 3.3 of the Results for further details on media collection timing optimization and **Figure** **3**). The medium was subsequently clarified through centrifugation and filtration to remove cellular debris, ensuring compatibility with downstream purification steps.

**Figure 4:**
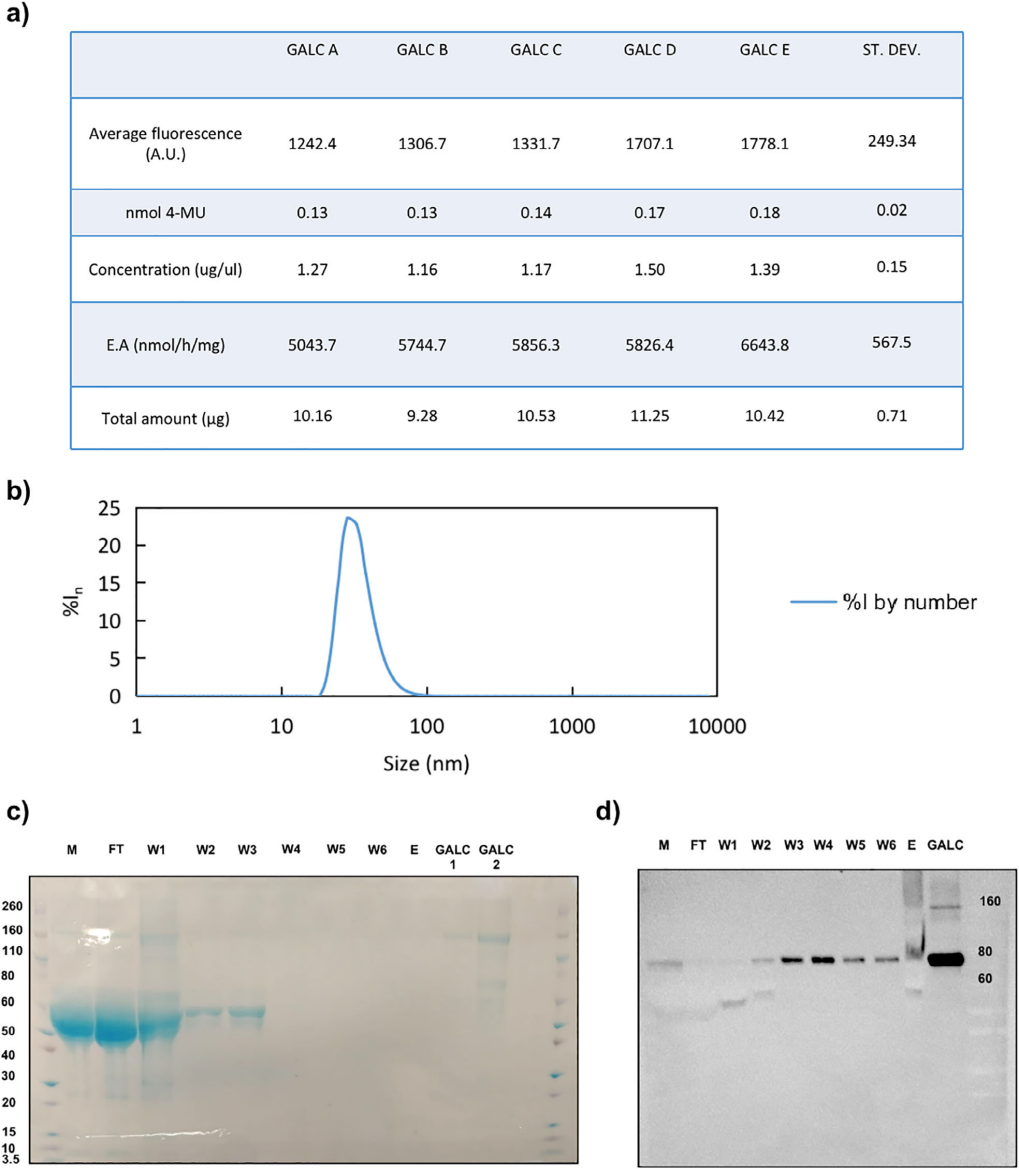
Characterization of the purified rm‐GALC. a) The table summarizes the characterization of five separate batches of rm‐GALC purified independently. The first row presents the average FL values obtained from the β‐gal assay. The second row reports the nmol of 4‐MU calculated by interpolation with the standard curve. The third row lists the protein concentration measured using the BCA assay. The fourth row shows the enzymatic activity, expressed as nmol of product per hour per mg of rm‐GALC used in the assay. The final row provides the total amount of rm‐GALC purified in each independent purification. b) DLS trace acquired on the rm‐GALC sample solution (percent intensity by number, %I_n_), indicating a monomodal distribution of molecular size and low polydispersion. c) The gel shows the SDS‐page profile for the fractions obtained from rm‐GALC purification. M = collected media; FT = flowthourght; W1–6 = wash 1–6; E = elution; GALC 1 – 2 = purified rm‐GALC charged in different amount. Contaminants are present up to W3, whereas the characteristic rm‐GALC bands are detected in GALC 1 and GALC 2. d) Western blot analysis of rm‐GALC in purification fractions. Western blotting performed on fractions obtained from rm‐GALC purification confirms the presence of rm‐GALC across all fractions, with higher concentrations observed in the purified rm‐GALC samples.

Affinity chromatography using Ni‐charged resin proved highly effective in isolating His‐tagged rm‐GALC, with six sequential washes using 25 mM imidazole buffer successfully removing contaminants. The elution step with 300 mM imidazole yielded highly enriched GALC fractions, as confirmed by SDS‐PAGE and Coomassie staining (**Figure** **4**c). Buffer exchange and concentration using 10 kDa cutoff filters not only replaced the elution buffer with the storage buffer but also ensured a final protein concentration of approximately 2 µg µL^−1^, suitable for both biochemical characterization and storage at −20 °C.

This workflow demonstrated excellent reproducibility across multiple purification batches, as evidenced by consistent yields and purity of rm‐GALC (See Section 3.4 of the Results and Figure 4a). Western blot analysis confirmed the identity of the purified enzyme (Figure 4d), and enzymatic activity assays showed that the purified protein retained its catalytic functionality. Moreover, DLS analysis indicated a homogeneous size distribution, further validating the quality of the preparation for downstream applications, including preclinical studies for ERT (Figure 4b). These results highlight the robustness of this workflow, providing a scalable and reliable method for the production of high‐quality recombinant GALC.

### 3.2 Enzymatic Assays Optimization

Figure S1a (Supporting Information) represents the chemical basis of the 4‐methylumbelliferyl‐β‐D‐galactopyranoside (4‐MU‐β‐gal) assay. Here, the GALC enzyme cleaves the 4‐MU‐β‐gal substrate, producing the fluorescent product 4‐methylumbelliferone (4‐MU), which serves as a direct measure of GALC enzymatic activity. This assay is specifically designed for purified GALC, as its use in lysed cells or tissue samples may yield inaccurate results due to potential cross‐reactivity with β‐galactosidase, which can also catalyze the reaction, potentially leading to inaccurate results. In contrast, Figure S1b (Supporting Information) illustrates the 6‐Hexadecanoylamino‐4‐methylumbelliferyl‐β‐D‐galactopyranoside (HMU‐β‐gal) assay, which is highly specific to GALC. This substrate, HMU‐β‐gal, is converted exclusively by GALC into the fluorescent product 6‐Hexadecanoylamino‐4‐methylumbelliferone (HMU). As a result, the HMU‐β‐gal assay can reliably measure GALC activity in diverse sample types, including lysed cells and tissues, without interference from other enzymes. Figure 1a,c presents the standard curve for 4‐MU fluorescence, essential for quantifying the enzymatic product in enzymatic assays. The fluorescence intensity is linear in the 0–30 nmol range, which was selected as the optimal window for accurate extrapolation of enzyme activity from fluorescence measurements.

Figure 1b,d evaluates the sensitivity and linearity of the 4‐MU assay in relation to GALC concentration. The fluorescence output is proportional to the amount of purified GALC in the 0–250 ng range, confirming that this range is optimal for enzymatic assays without risk of substrate saturation. This ensures reproducible and reliable quantification of GALC activity under purified conditions.

### 3.3 Optimization of Extracellular Medium Collection Timing

The timing of extracellular medium collection was optimized to maximize the recovery of enzymatically active recombinant murine GALC (rm‐GALC) from HEK‐293T cells cultured in roller bottles. Enzymatic activity in the extracellular medium was monitored at three time points: the day of culture establishment (T0), 5 days after establishment (T1), and 14 days after establishment (T2). These measurements were compared with those from unmodified HEK‐293T cells cultured under identical conditions.

As shown in Figure 3, no GALC enzymatic activity was detected in the extracellular medium of unmodified HEK‐293T cells at any of the tested time points. This confirms that HEK‐293T cells do not naturally secrete measurable amounts of GALC enzyme under these conditions. By contrast, cells engineered to overexpress rm‐GALC exhibited progressively increasing GALC activity in their extracellular medium. A statistically significant rise in enzymatic activity was observed at T1 compared to T0 (*p < 0.05; one‐way ANOVA, Dunnett's test). This increase continued, with activity levels peaking at T2 (****p < 0.0001; T2 versus T0).

These results demonstrate that rm‐GALC secretion is time‐dependent, with the highest enzymatic activity observed at T2, 14 days post‐culture establishment. Accordingly, T2 was selected as the optimal time point for extracellular medium collection, ensuring maximal recovery of enzymatically active rm‐GALC for subsequent purification steps.

### 3.4 Characterization of the Purified GALC

Figure 4 summarizes the results of the characterization of purified rm‐GALC, highlighting the enzymatic activity, protein concentration, and yield consistency across multiple purification batches.

In Figure 4a, the enzymatic activity of five different batches of rm‐GALC, obtained from independent purification procedures, is reported. The average enzymatic activity of purified rm‐GALC was determined to be 5822.98 ± 567.5 nmol h^−1^ mg^−1^, while the average protein concentration obtained from purification was 1.3 ± 0.15 µg µL^−1^, with a total yield per purification procedure of 10.33 ± 0.71 µg. Figure S2 (Supporting Information) further illustrates these results, providing a clear graphical representation of the enzymatic activity, protein concentration, and yield alongside their corresponding standard deviations.

Figure 4b presents the DLS characterization of purified rm‐GALC. The mean peak size measured was 24 ± 7 nm (FWHM), with a PDI of 0.2, indicating an open nonglobular conformation of the molecule in the medium used to prepare the solution. Moreover, we did not detect relevant peaks at higher size values, confirming the enzyme's homogeneity and excellent dispersion in the studied conditions.

The purification process of rm‐GALC was evaluated by SDS‐PAGE and Western blot, as shown in Figure 4c,d. The SDS‐PAGE analysis (Figure 4c) highlights the progressive removal of contaminants through the purification workflow. Significant contamination was observed in the extracellular medium (M), flow‐through (FT), and the first wash (W1), consistent with the presence of non‐specific proteins. However, subsequent washes (W2 and W3) showed a marked reduction in contaminants, with the final washes (W4, W5, and W6) displaying no detectable protein contamination, underscoring the effectiveness of the washing steps.

The elution fraction (E) did not exhibit visible protein bands in the gel, as expected, likely due to the dilution of rm‐GALC in the elution buffer. However, concentrated rm‐GALC samples (G1 and G2) revealed two distinct bands corresponding to molecular weights of approximately 70 and 150 kDa, matching the expected sizes for monomeric and dimeric forms of the GALC protein, respectively.

Western blot analysis (Figure 4d) confirmed the identity of these bands as GALC. The characteristic 70 kDa GALC band was faintly detected in the medium (M), flow‐through (FT), and W1 fractions, indicating the initial presence of GALC protein. In contrast, the W2–W6 fractions exhibited higher amounts of the 70 kDa GALC band, reflecting successfull enrichment of GALC during the purification process.

### 3.5 Rm‐GALC is Able to Promote Enzymatic Activity Recovery in KD Primary cells

**Figure 5:**
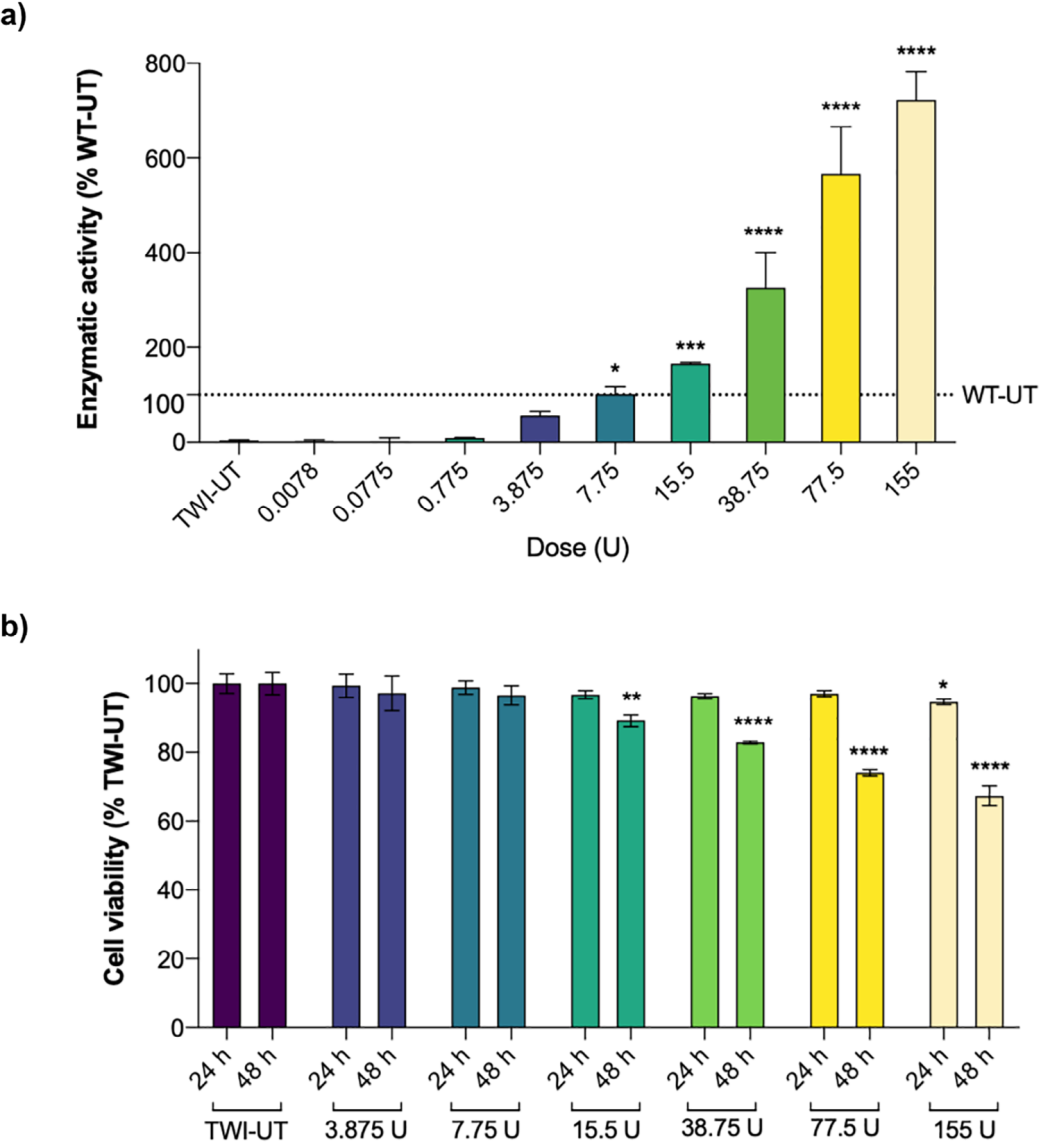
Characterization of enzymatic recovery and cell viability after in vitro ERT. a) Enzyme replacement therapy in vitro with rm‐GALC. Purified rm‐GALC was administered to KD primary cells at nine increasing concentrations: 0.0078 U, 0.0775 U, 0.775 U, 3.875 U, 7.75 U, 15.5 U, 38.75 U, 77.5 U, 155 U (enzymatic unit, U = nmol h−1). HMU‐β‐gal enzymatic assays were performed on cell lysates 4 h post‐administration. Results are reported in % of the WT‐UT activity. Dose of 7.75 U fully restores enzymatic activity to wild‐type (WT) levels (*p < 0.05 for Dose 7.75 U vs. TWI‐UT; One‐way ANOVA, Tukey's test). Dose 15.5 U 6 results in slightly higher enzymatic activity than WT cells (**p < 0.001 vs. WT‐UT; One‐way ANOVA, Tukey's test). Dose 38.75, 77.5, and 155 show significantly increased enzymatic activities compared to WT cells (****p < 0.0001 for Dose 38.75, 77.5 and 155 versus WT‐UT; One‐way ANOVA, Tukey's test). b) Cell viability assay conducted on TWI cells treated with Dose 3.875‐155 U 4–9 of rm‐GALC, measured 24 and 48 h post‐treatment. * *p* < 0.05 Dose 155 U 24 h versus TWI‐UT 24 h; ** *p* < 0.01 Dose 15.5 U 48 h versus TWI‐UT 48 h; **** *p* < 0.0001 Doses 38.75 U, 77.5 U, 155 U versus TWI‐UT 48 h; One‐way ANOVA, Dunnett's test. Data are reported as mean ± SEM; *n* = 3.

To assess the therapeutic potential of rm‐GALC, its ability to restore enzymatic activity was tested in primary TWI fibroblasts, a cellular model of KD. As illustrated in **Figure** **5**a, untreated TWI cells (TWI‐UT) exhibited nearly undetectable GALC enzymatic activity compared to untreated WT cells (WT‐UT; **** *p* < 0.0001, TWI‐UT versus WT‐UT, Student's t‐test). This confirms the severely deficient GALC activity in TWI cells, underscoring their suitability for in vitro ERT studies.

Thus, we performed in vitro ERT on TWI cells using nine increasing doses of GALC (0.0078 U; 0.0775 U; 0.775 U; 3.875 U; 7.75 U; 15.5 U; 38.75 U; 77.5 U; 155 U; enzymatic unit (U) = nmol/h). As shown in Figure 5a, the three lowest doses (0.0078 U, 0.0775 U, 0.775 U) do not induce a significant increase in GALC enzymatic activity in TWI cells. Dose 3.875 U, however, achieves approximately half of the enzymatic activity observed in WT cells, while Dose 7.75 U fully restores enzymatic activity to WT levels (*p* < 0.05 for Dose 7.75 versus TWI‐UT; One‐way ANOVA, Tukey's test).

Administration of Dose 15.5 results in enzymatic activity slightly exceeding that of WT cells (165%; *** *p* < 0.001 versus WT‐UT; One‐way ANOVA, Tukey's test).

The subsequent higher doses (Dose 38.75 U; 77.5 U; 155 U) result in significantly higher enzymatic activities than those of WT cells, reaching 326%, 566%, and 723% of WT activity, respectively (****p < 0.0001 for Dose 38.75 U; 77.5 U; 155 U versus WT‐UT; One‐way ANOVA, Tukey's test; Figure 5a).

### 3.6 Physiological Administration of rm‐GALC in KD cells Not Affects Cell Viability

To evaluate the impact of different doses of rm‐GALC on the viability of TWI cells, we conducted viability assays using doses that were associated with a significant increase in enzymatic activity (Doses 3.875‐7.75‐15.5‐38.75‐77.5‐155 U; Figure 5b). As expected, no reduction in cell viability was observed at rm‐GALC concentrations up to Dose 7.75 U, the dose that restored physiological GALC activity levels. These results suggest that the enzyme at therapeutic doses is well‐tolerated by TWI cells.

Interestingly, at slightly higher concentrations, a modest but statistically significant decrease in cell viability was observed at Dose 15.5 U (**p < 0.01, Dose 15.5 U versus TWI‐UT; one‐way ANOVA, Tukey's test). This trend became more evident as the dose increased further, with a marked reduction in cell viability detected at higher doses (****p < 0.0001 for Doses 38.75‐77.5‐155 U versus TWI‐UT; one‐way ANOVA, Tukey's test). These findings indicate a potential dose‐dependent cytotoxic effect of rm‐GALC at concentrations exceeding physiological activity restoration levels.

### 3.7 Dose‐Dependent Effects of GALC on the Autophagic Pathway

**Figure 6:**
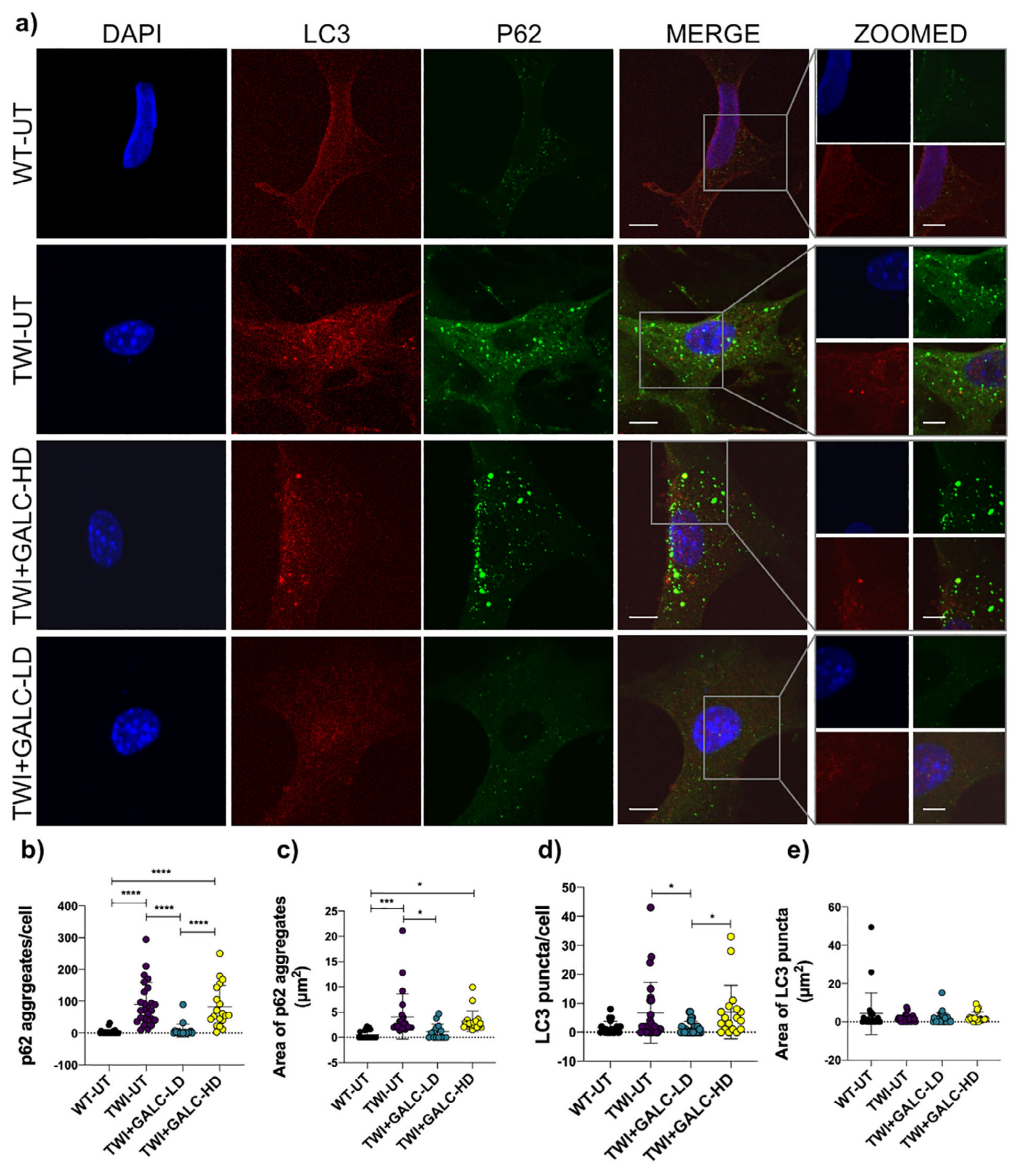
rm‐GACL‐mediated autophagy modulation in TWI (KD) primary cells. a) Immunohistochemistry staining of wild‐type untreated cells (WT‐UT), Twitcher untreated cells (TWI‐UT), Twitcher cells treated with a supra‐physiological dose of rm‐GALC (Dose 155 U; TWI+GALC‐HD), and Twitcher cells treated with the physiological dose of rm‐GALC (Dose 7.75 U; TWI+GALC‐LD). Cells are stained with anti‐LC3 (in red) and anti‐p62 (in green) antibodies, nuclei are stained with DAPI (in blue). Scale bar: 10 µm. A 8 X zoom is shown in the right part of the figure for each merged image. Scale bar zoomed image: 1.25 µm. **(b‐c)** Analysis of the number and average area of p62 aggregates per cell. b) Number of p62 puncta: **** *p* < 0.0001, TWI‐UT, and TWI+GALC‐HD versus WT‐UT, TWI+GALC‐LD versus TWI‐UT, and TWI+GALC‐HD versus TWI+GALC‐LD. c) Average area of p62 aggregates (µm^2^): * *p* < 0.05 TWI+GALC‐LD versus TWI‐UT and TWI+GALC‐HD versus WT‐UT. d,e) Analysis of the number and average area of LC3 puncta per cell. d) Number of LC3 puncta: * *p* < 0.05 TWI+GALC‐LD versus TWI‐UT and TWI+GALC‐HD versus TWI+GALC‐LD. e) Average area of LC3 puncta (µm^2^). Data are reported as mean ± SD, and compared using One way ANOVA (Tukey's multiple comparisons test). *n* = between 19 and 29 cells for each experimental group.

To investigate the effect of rm‐GALC administration on the autophagy pathway, we analyzed the expression and subcellular localization of two key autophagy markers, LC3‐II and p62, which we have previously reported as dysregulated in TWI cells.^[^ ^19^, ^20^, ^24^ ^]^ Specifically, we treated TWI cells with a physiological dose (Dose = 7.75 U) and a supra‐physiological dose (Dose = 155 U) of rm‐GALC to explore the potential impact of dose variation on autophagy regulation (**Figure** **6**a). Nuclei were stained in blue (first column), LC3 in red (second column), and p62 in green (third column). The fourth column displays merged images, and the fifth column shows zoomed‐in views. Cells treated with Dose 7.75 U (low dose) are referred to as TWI‐GALC‐LD, and cells treated with Dose 155 U (high dose) are referred to as TWI‐GALC‐HD (Figure 6a).

Consistent with previous studies from our group^[^ ^20^ ^]^ and others,^[^ ^21^ ^]^ we observed a significantly higher number of p62 aggregates in TWI‐UT cells compared to WT‐UT cells (****p < 0.0001, TWI‐UT versus WT‐UT; one‐way ANOVA, Tukey's test; Figure 6b). Similarly, the area of p62 aggregates was significantly larger in TWI‐UT cells than in WT‐UT cells (***p < 0.001, TWI‐UT versus WT‐UT; one‐way ANOVA, Tukey's test; Figure 6c).

Interestingly, treatment with the high dose of rm‐GALC (TWI+GALC‐HD) did not reduce the number or size of p62 aggregates compared to TWI‐UT cells (Figure 6b,c). However, treatment with the physiological dose of rm‐GALC (TWI+GALC‐LD) resulted in a significant reduction in the number of p62‐positive aggregates (****p < 0.0001, TWI+GALC‐LD versus TWI‐UT; one‐way ANOVA, Tukey's test; Figure 6b), bringing the levels close to those observed in WT‐UT cells. A similar trend was observed for the area of p62 aggregates (*p < 0.05, TWI+GALC‐LD versus TWI‐UT; one‐way ANOVA, Tukey's test; Figure 6c).

Regarding LC3 puncta, we observed trends consistent with those for p62 aggregates (Figure 6c). LC3 puncta were significantly increased in TWI‐UT cells compared to WT‐UT cells (*p < 0.05, TWI‐UT versus WT‐UT; one‐way ANOVA, Tukey's test; Figure 6c) and were significantly reduced in TWI+GALC‐LD cells compared to TWI‐UT cells (*p < 0.05, TWI+GALC‐LD versus TWI‐UT; one‐way ANOVA, Tukey's test; Figure 6c). However, no reduction in LC3 puncta was observed in TWI+GALC‐HD cells. Notably, the area of LC3 puncta did not vary significantly among the experimental groups (Figure 6d).

Finally, in the zoomed images (Figure 6a, last column), we clearly observed colocalization of p62 and LC3 in both TWI‐UT and TWI+GALC‐LD cells.

### 3.8 Suitability of GALC for Encapsulation in Polymeric Stabilized Reverse Micelles‐Based Nanocarriers

**Figure 7:**
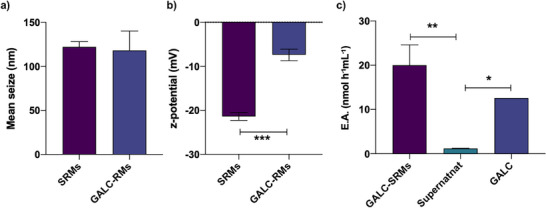
Rm‐GALC encapsulation into polymeric stabilized reverse micelle (SRM)‐based nanovectors. a) Hydrodynamic diameter (nm) of empty SRMs (SRMs) and rm‐GALC loaded SRMs (GALC‐SRMs). b) Zeta potential of empty SRMs and GALC‐SRMs. *** *p* < 0.001 SRMs versus GALC‐SRMs; Student's t‐test. c) Enzymatic activity of: GALC‐SRMs, the supernatant resulting from GALC loading into empty SRMs (supernatant), and GALC solution (GALC). ** *p* < 0.01 GALC‐SRMs versus supernatant; * *p* < 0.05 supernatant versus GALC; One way ANOVA with Bonferroni correction for selected pairs. Data are reported as mean ± SEM *n* = 3.

As shown in **Figure** **7**a, the produced SRMs exhibited a hydrodynamic diameter of 122 ± 6 nm (SEM). This value is consistently below 200 nm, thus, it is in an optimal size range for cellular uptake and brain delivery, which are crucial for therapeutic applications targeting neurodegenerative diseases like KD.^[^ ^7^, ^28^, ^29^ ^]^ Additionally, the surface zeta potential of the RMs was −21 ± 0.9 mV (Figure 7b), meeting the criteria for administration.^[^ ^28^, ^30^ ^]^

Upon loading with rm‐GALC, the SRMs demonstrated substantial enzymatic activity, reaching approximately 20.4 ± 7.6 nmol h^−1^ mL^−1^, corresponding to 25.5 ± 9.6 nmol h^−1^ mL^−1^ of SRMs (Figure 7c), while the residual activity in the supernatant was 1.2 ± 0.1 nmol h^−1^ mL^−1^, significantly lower than the starting activity (12.6  nmol h^−1^ mL^−1^). The GALC‐SRMs size did not significantly change (118 ± 22 nm), while the z‐potential slightly increased (−7.4 ± 1.3 mV). This result highlights the efficiency of the encapsulation process and confirms that rm‐GALC retains its enzymatic activity within the SRMs.

## 4 Discussion

This study provides a comprehensive framework for the purification, characterization, and therapeutic evaluation of recombinant murine GALC (rm‐GALC), addressing critical challenges in ERT for KD. The results contribute to a deeper understanding of the optimized production of rm‐GALC, its biochemical and functional properties, and its dose‐dependent effects in the pathological context of KD.

First, the workflow developed here for rm‐GALC purification (Figure 2) reliably produces pure and stable rm‐GALC, suitable for downstream applications. Key steps, such as pre‐centrifugation and optimized washing and elution conditions, efficiently remove contaminants while maintaining enzyme integrity. The final product, stored under conditions that preserve enzymatic activity, was validated through SDS‐PAGE and DLS analyses, confirming both purity and enzymatic efficiency. This robust method provides a reproducible approach for rm‐GALC production, and its detailed description in this article may serve as a valuable resource for advancing research on KD or for the expression of eukaryotic enzymes more broadly.

Additionally, we optimized the timing for medium harvesting, as shown in Figure 3. Temporal analysis of enzymatic activity in the extracellular medium of HEK‐293T cells revealed that rm‐GALC secretion increases significantly over time, peaking 14 days after culture establishment. This result guided the selection of the optimal collection timing, maximizing enzyme yield during each purification. These insights ensure efficient resource utilization and provide guidance for cultural practices in large‐scale production.

For the characterization of the enzymatic activity of the purified rm‐GALC in different environments, we optimized two enzymatic assays utilizing 4‐MU‐β‐gal and HMU‐β‐gal substrates (Figure 2 and Figure S1, Supporting Information). The 4‐MU‐β‐gal assay provided a reliable means to measure enzyme activity in purified preparations, while the HMU‐β‐gal assay extended this capability to lysed cells and tissue samples.

Subsequently, we assessed the capacity of rm‐GALC to restore enzymatic activity in primary TWI cells four hours post‐administration (Figure 5). Lower doses (0.0078 U, 0.0775 U, and 0.775 U) showed no significant effect on intracellular GALC activity. Dose 3.875 U achieved approximately 50% of WT enzymatic activity. Notably, Dose 7.75 U fully restored activity to WT levels, while Dose 15.5 U exceeded WT activity. Higher doses (38.75 U, 77.5 U, and 155 U) resulted in supraphysiological activities, reaching 326%, 566%, and 723% of WT levels, respectively. These results are consistent with our previous study, where the doses of 0.75, 1.5, 3, and 6 U of rm‐GALC were used as controls to deliver the same enzymatic units of free rm‐GALC as those delivered with nanoparticles, and in those conditions, none of the doses were able to restore WT activity in TWI cells.^[^ ^20^ ^]^

Interestingly, instead, the dose of 6 U was found to be efficient in restoring the WT enzymatic activity if delivered by targeted nanoparticles.^[^ ^20^ ^]^

Here, we identified the dose of free rm‐GALC capable of restoring WT activity and highlighted the enzyme's ability to surpass physiological levels at higher doses.

To assess the susceptibility of KD cells to rm‐GALC administration in terms of cell viability, we measured cell survival at the doses previously found to induce a significant increase in intracellular GALC activity (Doses 3.875‐155 U). Viability assays confirmed that rm‐GALC is well‐tolerated at therapeutic doses, with no cytotoxic effects observed up to the dose required for physiological activity restoration (Dose 7.75). However, higher doses induced a dose‐dependent decrease in cell viability, suggesting potential cytotoxicity at excessive concentrations. These results emphasize the importance of dosing precision to maximize therapeutic efficacy while avoiding adverse effects.

Previously reported data further demonstrate that supraphysiologic GALC activity, caused by inherited genetic deficiency or forced gene expression in KD patient cells and disease models, induces alterations in the intracellular content of the bioactive GALC downstream products, ceramide and sphingosine, affecting hematopoietic stem/progenitor cell (HSPC) survival, functionality, and the stem cell niche.^[^ ^31^ ^]^

Interestingly, recent evidence also highlights a pro‐oncogenic role of GALC overexpression in melanoma. GALC overexpression was shown to decrease ceramide levels and increase the tumorigenic activity of human melanoma A2058 cells, while GALC downregulation exerted the opposite effect. Accordingly, a progressive increase in GALC expression has been demonstrated from common nevi to stage IV human melanoma samples.^[^ ^32^ ^]^

Thus, in the context of ERT for KD, the results presented here, in line with existing literature, emphasize the critical importance of carefully fine‐tuning the administered dose to achieve optimal therapeutic efficacy while minimizing potential adverse effects.

Additionally, we investigated the impact of rm‐GALC on the modulation of the autophagic pathway. In previous studies, our group demonstrated dysregulation of the autophagic pathway in both in vitro models^[^ ^19^, ^20^ ^]^ and the in vivo model of KD, the TWI mouse.^[^ ^20^, ^22^, ^24^, ^33^ ^]^

Specifically, we identified dysregulation in key autophagy markers in KD models, including LC3, p62, Beclin‐1, and autophagy‐related protein 5 (ATG5).^[^ ^20^, ^22^ ^]^ We observed p62‐tagged protein aggregates in the brain of KD mice and increased p62 levels in the KD sciatic nerve, suggesting an impaired ability of KD cells to effectively clear p62‐tagged waste molecules. These findings point to a possible blockage in the autophagy flux in KD.^[^ ^20^, ^21^ ^]^ Furthermore, we showed that the autophagy inducer Rapamycin (RAPA) can partially restore the WT phenotype in KD primary cells^[^ ^20^ ^]^ and in the TWI mouse,^[^ ^24^ ^]^ reducing the number of p62 aggregates in both KD cells and neural tissues. In vivo results from RAPA treatments in the TWI mouse were also confirmed by the group of Lin and colleagues.^[^ ^34^ ^]^

Autophagy dysregulation in KD has also been reported by other research groups. Ribbens et al. found increased levels of LC3‐B in a novel KD cell model, suggesting that this could either indicate autophagy activation or a disruption in the autophagic process due to lysosomal dysfunction caused by the primary GALC deficiency.^[^ ^35^ ^]^ In a subsequent study, Rebiai et al. observed impaired fusion between autophagosomes and lysosomes in neural precursor cells derived from two novel mouse models of KD, which mimic both early‐ and adult‐onset forms of the disease.^[^ ^36^ ^]^ Notably, Papini et al.^[^ ^37^ ^]^ recently demonstrated that AKT‐mediated inhibitory phosphorylation of Beclin‐1 and the formation of the BCL2‐Beclin‐1 complex contribute to the reduced formation of autophagosomes in fibroblasts from KD patients, independent of psychosine accumulation.

**Figure 8:**
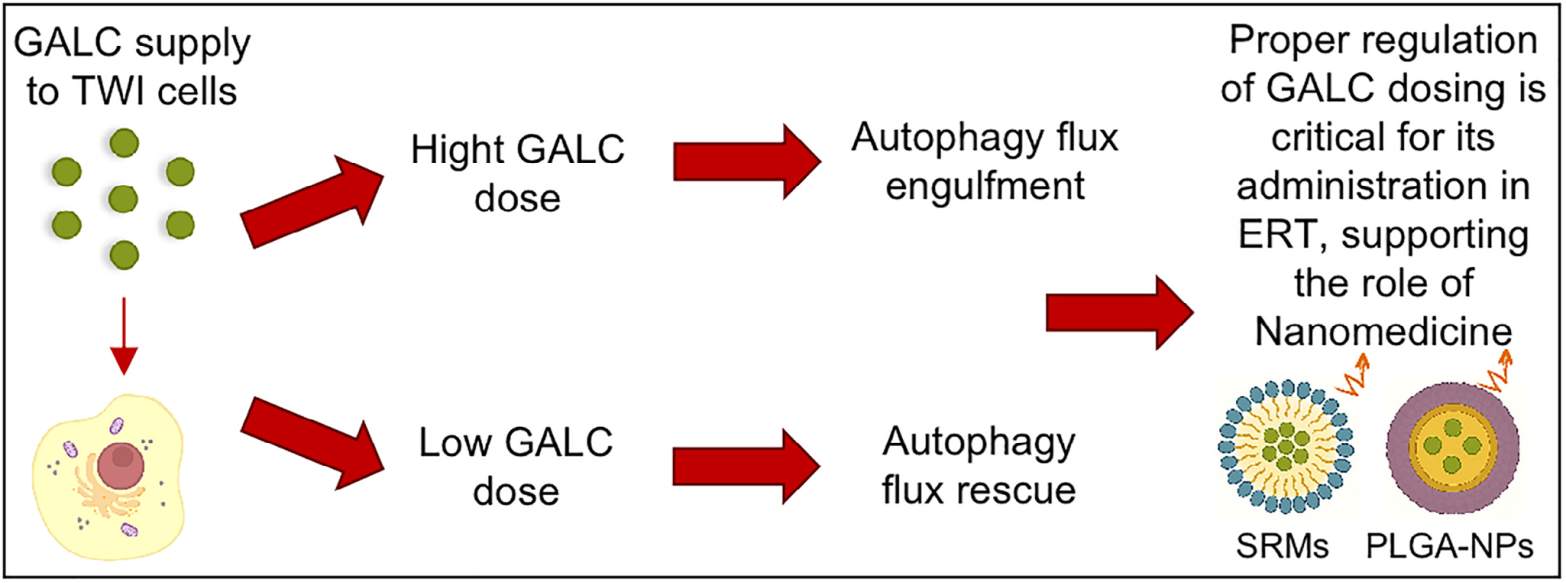
Effect of rm‐GALC on autophagy regulation. This schematic illustrates the hypothesized impact of rm‐GALC dosing on autophagy regulation. Administration of a supra‐physiological dose may overwhelm the autophagy flux, impairing the proper degradation of materials within the pathway. Conversely, our results indicate that administering a physiological dose of rm‐GALC sufficient to restore normal enzymatic activity supports the recovery of autophagy flux. This promotes the clearance of abnormally accumulated materials typically observed in TWI cells. These findings underscore the importance of precise dosing in therapeutic strategies aiming to restore GALC activity and normalize cellular processes in KD tissues, strongly supporting the role of nanomedicine‐based ERT strategies. A schematic nanovector representation is shown: stabilized reverse micelle (SRMs; right) and polymeric nanoparticles (PLGA‐NPs; left). RmGALC = recombinant murine Galactosylceramidase; TWI = Twitcher; ERT = enzyme replacement therapy.

In this study, we revealed for the first time a dose‐dependent effect of GALC administration on the autophagic accumulation of p62‐tagged aggregates in KD cells. Specifically, the administration of a supra‐physiological dose of rm‐GALC had no effect on the number or area of p62 aggregates. This suggests that an excessive dose of GALC may impair the autophagic flux, preventing the proper degradation of p62‐tagged materials. Conversely, the administration of a physiological dose (defined as the dose that restores WT enzymatic activity) significantly reduced both the number and area of aggregates (Figure 6a–c), bringing these values to levels comparable to those observed in WT cells. A schematic representation of this concept is shown in **Figure** **8**.

Consistently, the number of LC3 puncta also decreased in KD cells treated with the physiological dose of rm‐GALC, aligning with the observation that p62‐tagged autophagy cargo fuses with autophagosomes to enable the degradation of tagged materials.^[^ ^38^ ^]^ In support of this, we observed clear colocalization of p62 with LC3 in rm‐GALC‐treated cells, indicating that this stage of autophagy is properly carried out in KD cells and that rm‐GALC administration does not impair it.

Overall, the findings obtained from this work underline the critical need to carefully regulate GALC dosing during its administration in ERT. Supra‐physiological doses of GALC, in fact, led to a decrease in cell viability and impaired the autophagic flux, hindering the proper recycling of waste molecules. Interestingly, these findings strongly support the use of controlled delivery systems for GALC to enable a fine‐tuned release of the enzyme, ensuring precise concentration regulation and prolonged persistence in biological fluids (Figure 8).

In this context, we provided evidence for the ability of an inverse micelle‐based nanomedicine approach^[^ ^23^ ^]^ to efficiently encapsulate GALC in an enzymatically active formulation, suitable for brain delivery (Figure 7).

Promising delivery tools include biocompatible and biodegradable options, such as polymeric nanoparticles or micelles. Polymeric nanoparticles have already demonstrated efficacy in restoring GALC activity in both in vitro and in vivo models of KD,^[^ ^20^ ^]^ while stabilized reversed micelles represent a promising avenue for exploration due to their ability to efficiently encapsulate hydrophilic compounds [ref. 23; Figure 7].

Altogether, nanovector‐based ERT represents a promising approach for precisely controlling the dose of therapeutic enzymes, addressing a critical challenge in treating diseases like KD. By utilizing nanocarriers, which can be tailored in terms of size, surface charge, and composition, it is possible to achieve controlled, sustained release of the enzyme, ensuring consistent and stable therapeutic concentrations over time.^[^ ^39^, ^40^ ^]^ This approach not only prevents the issues associated with suboptimal enzyme levels, such as insufficient therapeutic efficacy, but also mitigates the risks of toxicity that may arise from supraphysiological enzyme activity. Furthermore, sustained release via nanovectors decreases dosing frequency, improving treatment convenience.^[^ ^38^ ^]^ As a result, this strategy could enhance both the safety and effectiveness of ERT, offering significant potential to optimize treatment outcomes for KD and other LSDs. The ability to fine‐tune enzyme doses also opens the door for better management of enzyme‐related side effects, ultimately improving the long‐term sustainability of ERT.^[^ ^33^ ^]^

In conclusion, this study provides an important tool for the production of recombinant murine GALC (rm‐GALC), presenting a detailed and efficient protocol for the enzyme's purification and characterization, which was previously lacking in the literature in such detail. Additionally, for the first time, we highlight the effects of different GALC dosing on crucial factors, such as cell viability and autophagy. These findings address critical challenges in the optimization of ERT for KD and support the advancement of targeted delivery strategies based on nanovectors.

## Conflict of Interest

The authors declare no conflict of interest.

## Author Contributions

Ambra Del Grosso: Writing – review & editing, Writing – original draft, Methodology, Investigation, Funding acquisition, Formal analysis, Data curation, Conceptualization. Marco Cecchini: Writing – review & editing, Supervision, Resources, Project administration, Investigation, Funding acquisition, Formal analysis, Data curation, Conceptualization. Sara Carpi: Methdodology, Investigation. Laura Colagiorgio: Methdodology, Investigation. Miriam De Sarlo: Methodology, Investigation. Mariacristina Gagliardi: Methodology, Investigation.

## Supporting information



Supporting Information

## Data Availability

The data that support the findings of this study are openly available in ZENODO at https://doi.org/10.5281/zenodo.14548070, reference number 14548070.
