# Supplementary material for: Investigating the Cellular Effects of GALC Dosing in Enzyme Replacement Therapy for Krabbe Disease Supports the Role of Nanomedicine
Source: Adv Biol (Weinh). 2025 Jul 1;9(9):e00147. doi: 10.1002/adbi.202500147 (PMC12447118; doi:10.1002/adbi.202500147)
Supplement: Supplementary file 1 — Supporting Information [file ADBI-9-e00147-s001.docx]

**Supplementary material**

**
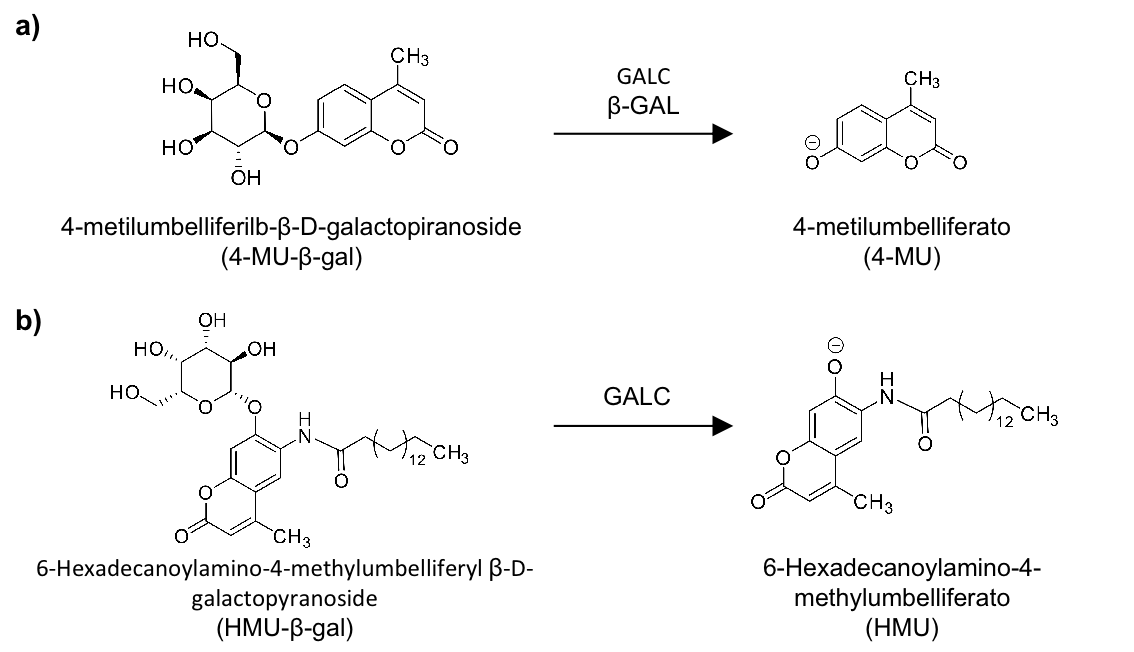
**

**Figure S1. (a)** 4-MU enxymatic assay**:** the glycosidic bond of the substrate 4-methylumbelliferyl-β-D-galactopyranoside (4-MU-βGal) is cleaved by the GALC enzyme, resulting in the formation of the product 4-methylumbelliferone (4-MU), which is fluorescent at basic pH. **(b)** HMU enzymatic assay: the glycosidic bond of the substrate 6-hexadecanoylamino-4-methylumbelliferyl b-D-galactopyranoside (HMU-β-gal) is cleaved by the GALC enzyme, resulting in the formation of the product 6-hexadecanoylamino-4-methylumbelliferato (HMU), which is fluorescent at basic pH.


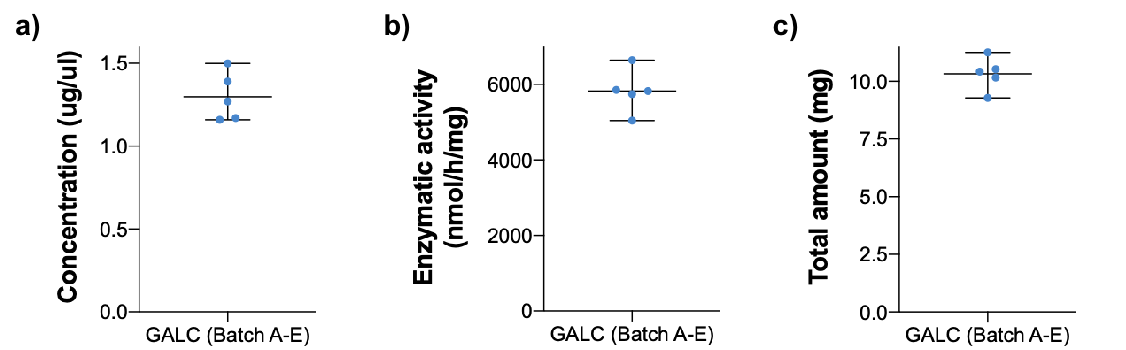


**Figure S2.** Characterization of purified rm-GALC. The graphs summarizes the characterization of five separate batches of independently purified rm-GALC. The first graph **(a)** shows the average enzyme concentration (μg/μl), the second **(b)** the enzymatic activity (nmol/h/mg), and the third **(c)** the total amount (mg), with the respective ranges.
